# Prenatal Exposure to Famine and Risk for Development of Psychopathology in Adulthood: A Meta-Analysis

**DOI:** 10.26502/jppd.2572-519X0077

**Published:** 2019-10-18

**Authors:** K. Dana, J. Finik, S. Koenig, J. Motter, W. Zhang, M. Linaris, J. C. Brumberg, Y. Nomura

**Affiliations:** 1 Queens College, CUNY, Psychology, New York, NY, USA; 2 The Graduate Center, CUNY, New York, NY, USA; 3 Graduate School of Public Health and Health Policy, CUNY, Epidemiology & Biostatistics, New York, NY, USA; 4 Icahn School of Medicine at Mount Sinai, Psychiatry, New York, NY, USA; 5 Memorial Sloan-Kettering Cancer Center, Department of Psychiatry and Behavioral Sciences, New York, NY, USA; 6 New York State Psychiatric Institute, New York, NY, USA; 7 Macaulay Honors College at Queens College, CUNY, New York, NY, USA

**Keywords:** Prenatal famine, Developmental psychopathology, Affective disorders, Psychotic disorders, Personality disorders

## Abstract

Prenatal famine, resulting in intrauterine malnutrition, impacts offspring psychopathology later in adulthood. In addition, the specific impact of intrauterine malnutrition of different psychopathology differs by the timing of the exposure. Using a meta-analysis, the current study assessed the specific risk of developing affective, psychotic, and personality disorders. Studies were identified using PubMed and PsycINFO. Studies met the following criteria for inclusion in the analysis: availability in peer-reviewed English journals, use of human subjects, prenatal exposure to famine, and psychopathology in adulthood defined by diagnostic criteria as an outcome. Fixed effect relative risks (RRs) were calculated for affective, psychotic, and personality domains. Furthermore, timing of exposure was assessed as an effect modifier in our analysis, defined by the index trimester at the height of famine. Our meta-analysis found that adults exposed *in utero* during the 1^st^ trimester were at a significant increased risk of psychotic disorders (RR=1.46, 95% CI=1.08, 1.97, p=0.014), and personality disorders (RR=2.31, 95% CI=1.36, 3.92, *p*=0.002). Those exposed during the 2^nd^ trimester were at a significant increased risk of affective disorders (RR=1.45, 95% CI=1.22, 1.72, *p*<0.0001), and psychotic disorders (RR=1.46, 95% CI=1.13, 1.89, p=0.004). Similarly, those exposed in the 3^rd^ trimester were at a significant increased risk of affective disorders (RR=1.33, 95% CI=1.13, 1.57, *p*=0.0001), and psychotic disorders RR=1.47, 95% CI=1.10, 1.97, p=0.010). Our findings suggest that there is differential risk across the different domains of psychopathology by trimester of exposures. This meta-analysis underscores the need for further investigation into the mechanisms underlying prenatal maternal nutrition and offspring psychopathology where magnitude of elevated risk differs by the exposure timing during pregnancy.

## 1. Introduction

Features of neurobehavioral functioning in the growing child do not originate at birth. Rather, critical periods may exist in pregnancy, during which the determinants of parturition are especially vulnerable to the effects of prenatal stress and other complications [1, 2]. During these periods, a developing fetus must rely solely on the pregnant mother for nutrients. Malnutrition *in utero* have been consistently associated with negative consequences for the growth, development, and overall health of affected offspring in both human and animal models [3].

## 2. Malnutrition *in utero* in Preclinical Research

A wealth of animal research suggests that a fetus can be “programmed” *in utero* for subsequent development and health following a stressful or taxing period, such as malnutrition [4], and these effects may be evident in adulthood [3]. One of the obvious advantages of animal models is an experimental control over variables related to malnutrition, including timing of exposure, percentage of calorie reduction, and the mother’s physical enviromnent. One way in which fetal programming has been evaluated in animal models is through diet manipulation whereby pregnant mothers were given a low-protein diet to simulate stressful periods in which food intake is diminished. A series of studies [5-7] established critical time periods. Specifically, rats which were fed a low-protein diet prior to conception had pups that grew more rapidly than controls from days 14-19. However, after day 19, the pups grew more slowly than controls and were significantly smaller than controls at birth. When the low-protein diet was given to mothers only after conception, pups grew significantly faster than controls until day 14, but were significantly smaller than controls after day 19. These changes may be explained in part by earlier findings in rats which showed that mothers who were fed altered protein supplies while pregnant gave birth to offspring with slowed changes in cell number in tissues such as the pancreas, elevated adult blood pressure, and traits indicative of later development of renal disease [7, 8].

## 3. Malnutrition *in utero* in Human Studies

Due to ethical constraints, variables cannot be manipulated in human studies as freely as in animal research. As such, cohort studies of significant famines are leveraged as natural experiments to evaluate the downstream effects of food intake during pregnancy. The Dutch famine study, which examined the impact of the Dutch Hunger Winter that brought a five-month period of acute starvation during the final months of World War II between 1944 and 1945, is one of the best-known studies in this field [9]. It took place in the German-occupied western part of the Netherlands when a German blockade cuts off food and fuel shipments. More than 20,000 deaths were attributed to the famine, which was alleviated in May 1945 when the Netherlands was liberated by the Allied forces. Subsequent famines, including the Chinese famine that spanned from 1959-1961 [10], similarly provide opportunities to examine the effects of malnutrition *in utero*. The Chinese famine occurred as a result of the Great Leap Forward, a campaign by the Communist Party of China to transform the country from an agrarian economy to a socialist society. During this period, a combination of flawed agricultural practices, limited cultivated land, and severe weather led to a devastating famine, with varying dates of onset and relief from the famine. The Anhui province was among the most severely impacted, with mass starvation beginning in the spring of 1959 and persisting until early 1961. Cohort research from this province has contributed to the *in utero* famine literature, allowing researchers to replicate the Dutch famine study findings in a different culture and region. Although the famine was a disaster for the thousands who died and many more who suffered, it provided a wealth of information regarding the impact of insufficient nutrition on maternal, fetal, and child health in a human population.

While other significant famines have occurred worldwide, a lack of reliable data has made it difficult to study them using precise epidemiological research [10]. As a result, famines that have occurred in the past century have not been examined in the context of famine research. Despite these limitations, famine data, particularly from the Dutch Hunger Winter and the Great Leap Forward in China provide convincing evidence that malnutrition *in utero* increases offspring risk of negative long-term physical and mental health consequences.

## 4. The Effect of Malnutrition *in utero* on Mental Health

Findings regarding mental health outcomes in connection with famine have been robust, though variable depending on a range of factors, including trimester, duration of exposure, and offspring sex. Overall, mental health problems and a lower quality of life in adulthood have been associated with *in utero* exposure to the famine, regardless of the trimester of exposure [11]. Another study showed that women exposed to a famine *in utero* were four times as likely to develop a mental disorder in adulthood compared with those born after the famine [12]. Notably, this effect was not found in men. With cognitive functioning, no notable difference was found between men exposed to a famine *in utero* and those who were unexposed at the age of 18. A subsequent study in men aged 56-59, revealed that cognitive deficits in selective attention developed in late adulthood [13]. As a selective attention is a cognitive ability that typically declines with age in a normative sample, the authors speculated that the greater deficits found in those exposed to famine may be related to an early manifestation of accelerated cognitive aging.

## 5. Timing of Exposure and Specific Mental Health Sequalae

The effects of prenatal famine on mental health outcomes appear to be moderated by the timing of *in utero* exposure. 1^st^ trimester exposure has been associated with increased stress reactivity, defined by heightened physiological responses following exposure to a stressful stimulus [11]. Adults exposed to famine during their 1^st^ trimester had increased blood pressure responses to psychosocial stress, suggesting heightened stress appraisal when compared to controls [11]. It is possible that differential sequalae by timing of exposure could lead to deficits in different domains of mental health problems.

### 5.1 Affective disorders

Various affective disorders, including affective psychosis, unipolar depression, bipolar disorder, and neurotic depression [14, 15] have been associated with malnutrition *in utero*. Affective psychosis, a diagnosis in the DSM III, is characterized by a severe disturbance of mood and at least one psychotic symptom (e.g., delusions) [16]. Bipolar disorder is a mood disorder characterized by alternating periods of depressive and manic behaviors [17]. In contrast, unipolar depression consists of depressive symptoms, without manic episodes. Neurotic Depression, an ICD-9 criterion [18], is characterized by depression with a disproportionate response to a disturbing experience. In a study exploring affective disorders in adults exposed to the Dutch famine, 2nd trimester exposure was associated with significantly increased relative risk (RR) of developing affective psychosis in adulthood in males, but not females [14]. However, no association was found between *in utero* famine exposure and neurotic depression.

In a subsequent study, those exposed during the 3^rd^ trimester were at significantly increased risk of a major affective disorder requiring hospitalization in adulthood for both men and women [15]. 2^nd^ trimester exposure remained significant for men, but only marginally significant for women. For unipolar depression, there was a significantly increased risk with 2^nd^ and 3^rd^ trimester exposure, whereas only a marginally significant increased risk for bipolar depression was observed for 2^nd^ trimester exposure in both men and women. Taken together, these results indicate that the 2^nd^ trimester may be the most critical time for ‘programming’ of downstream affective disorders, and that men may be more vulnerable to the harmful effects of maternal malnutrition during this period.

### 5.2 Psychotic disorders

Psychotic disorders, including schizophrenia, are characterized by disorganized thoughts and behaviors, delusions, hallucinations, and negative symptoms [16]. Susser and his colleagues examined the frequency of hospitalized patients who met criteria for schizophrenia in adulthood in a cohort exposed to the Dutch famine [19, 20]. Men and women exposed during the 1^st^ trimester (born October 15-December 31 1944) had a two-fold increased risk for schizophrenia. Similarly, an approximately two-fold increase in risk for schizophrenia was found among those born during the height of the Chinese famine in 1960 (RR=2.30) and 1961 (RR=1.93).

Conception during the height of the Chinese or Dutch famines increased schizophrenia risk when compared with pre-and post-famine cohorts. However, findings regarding schizophrenia risk differed between the Dutch famine and the Chinese famine in rural settings [21]. In rural China the post-famine cohort had the highest risk of developing schizophrenia when compared with the pre-famine and during-famine cohort. Furthermore, there was no significant difference between the unexposed (pre-famine group) and exposed cohorts in risk for schizophrenia in rural areas. Authors speculate that systematic bias by the high mortality rate in rural areas may have contributed to the observed findings. It is possible that selection bias in the famine cohort led to the survival of only those least susceptible and most resilient to disease.

Affective psychosis falls into the classification of psychotic disorders in this paper. Schizophrenia spectrum personality disorders, a continuum of symptoms encompassing schizoaffective disorder, schizophrenia, schizoid, and schizotypal personality disorders, have been classified under the psychotic and personality domain, due to the range of psychotic symptoms that often accompany the disorders [20].

### 5.3 Personality disorders

Antisocial personality disorder (ASPD), characterized by a pattern of disregard for and violations of the rights of others, manifested through repeated illegal acts, dishonesty, aggressiveness, and lack of remorse [17], was assessed as a potential outcome following famine exposure [22]. In a cohort of 18-year-old men exposed to the Dutch famine during their 1^st^ and/or 2^nd^ trimester, men had a significantly higher risk of developing ASPD by the age of 18 when compared to controls exposed during the 3^rd^ trimester. In the same study, men exposed during the 1^st^ and/or 2^nd^ trimester had a 2-fold increased risk of developing schizophrenia spectrum personality disorders (schizotypal, schizoid, paranoid, and avoidant personality disorders) as compared to 3^rd^ trimester controls [20].

While there has been substantial research on the physical health effects of prenatal malnutrition, to date, no study has combined the findings of famine research as it relates to psychopathology in a comprehensive analysis. A meta-analysis on affective, psychotic, and personality disorders may yield a more global and comprehensive understanding of prenatal famine risks on offspring mental health. This analytic method affords the opportunity to assess the reliability among studies and compare specific diagnoses within the context of the broader diagnostic category. Furthermore, the differential risk for disorders by trimester can be explored, so that trimester-specific effects can be elucidated.

## 6. Methods

### 6.1 Informational sources

**Figure 1:**
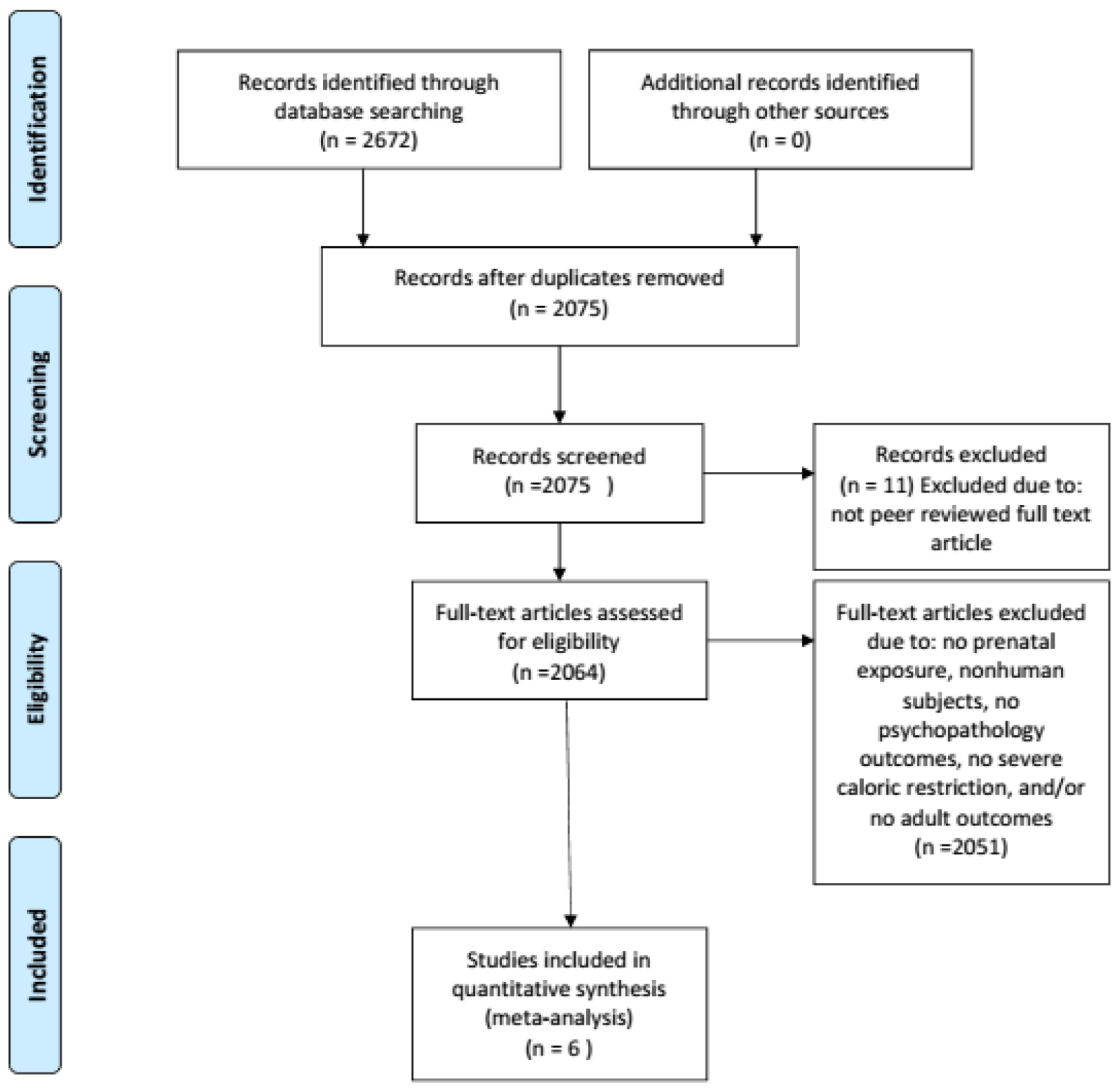
Flowchart illustrating literature search and exclusion process.

Eligibility criteria were as follows: use of human subjects, prenatal exposure to famine and severe caloric restriction exposure, psychopathology in adulthood defined by diagnostic criteria as an outcome, and availability in a peer-reviewed English journal (see Figure 1). The search terms used included: prenatal malnutrition and prenatal famine exposure. Studies were identified by using PubMed and PsycINFO from the first available date until September 30, 2016. Eligibility assessment was performed in a non-blinded standardized manner by the first author and was revised by input from additional authors. To identify additional studies that may have been appropriate for analysis, references from all relevant literature revealed by database searches were hand-searched. Review papers, commentaries, case reports, and book chapters were excluded. Articles assessing individual symptoms (i.e., depressive symptoms) rather than diagnostic outcomes such as Bipolar Disorder were excluded from this analysis. Only studies that assessed outcomes for adults who were exposed to famine *in utero* were included in the study. Studies using animal subjects were excluded. Studies that only assessed postnatal exposure to famine rather than prenatal exposure were also excluded. Six peer-reviewed articles met our final criteria, some of which included multiple analyses for different disorders. For these articles, multiple mental health outcomes in an article were analyzed as separate “studies” so that specific psychopathology could be assessed.

In order to capture a comprehensive picture of the effect of exposure on disorders whose symptomatology may not pertain exclusively to one domain, two disorders were included for analysis in multiple domains; affective psychosis was included in both the affective and psychotic domains, and schizoid personality disorder was included in both the psychotic and personality domains.

### 6.2 Statistical analyses

**Figure 2:**
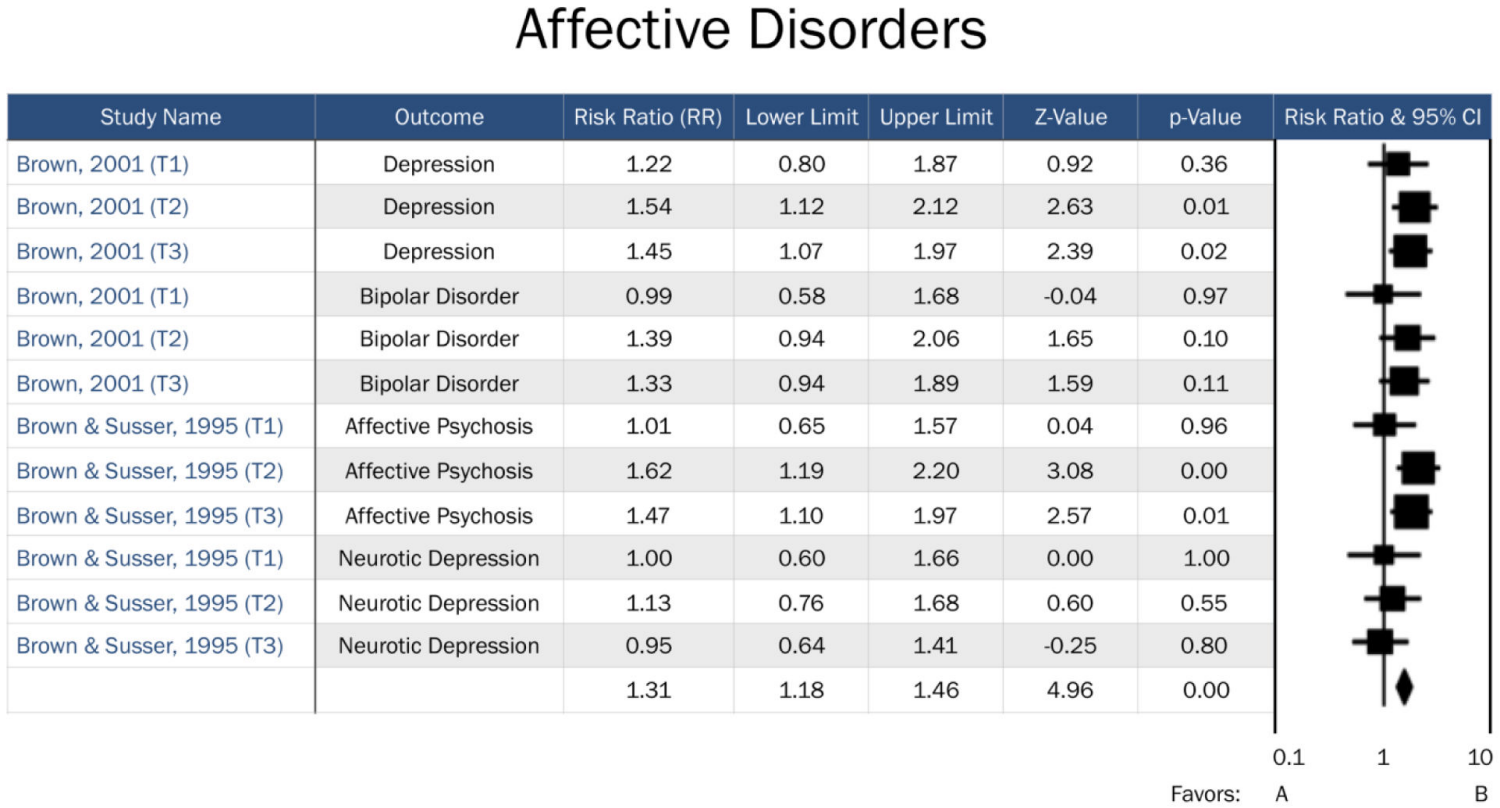
Forest plot of all studies in the affective disorders domain. T1=Trimester 1, T2=Trimester 2, T3=Trimester 3. Favors A=negative association between prenatal famine exposure and affective disorders in adulthood. Favors B= positive association between prenatal famine exposure and affective disorders in adulthood.

**Figure 3:**
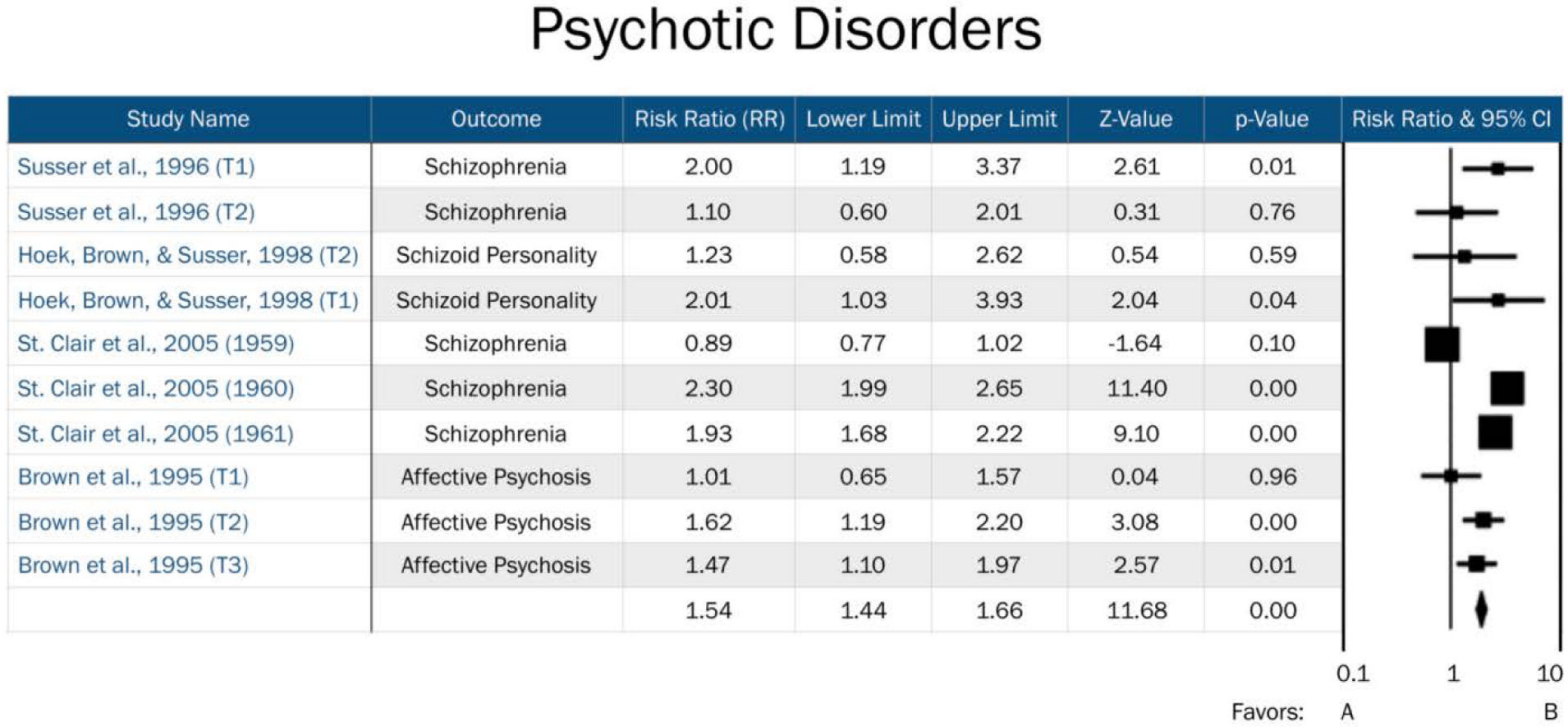
Forest plot of all studies in the psychotic disorders domain. T1=Trimester 1, T2=Trimester 2, T3=Trimester 3. Year of exposure included when no trimester data available. Favors A=negative association between prenatal famine exposure and psychotic disorders in adulthood. Favors B= positive association between prenatal famine exposure and psychotic disorders in adulthood.

**Figure 4:**
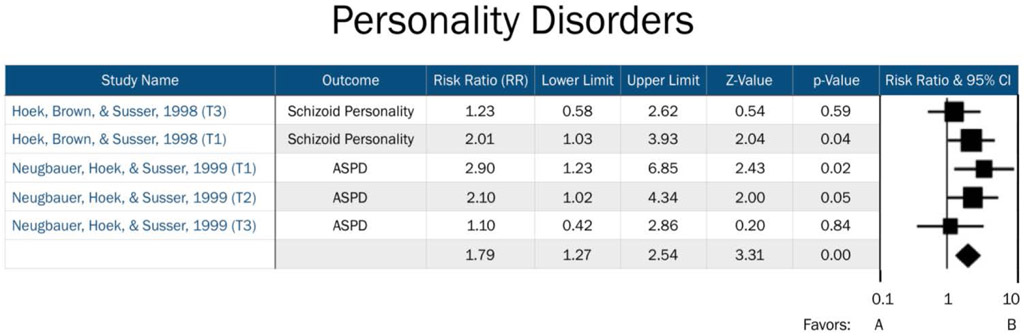
Forest plot of all studies in the personality disorders domain. T1=Trimester 1, T2=Trimester 2, T3=Trimester 3. Favors A=negative association between prenatal famine exposure and personality disorders in adulthood. Favors B=positive association between prenatal famine exposure and personality disorders in adulthood.

Comprehensive Meta-Analysis (CMA) Version 3.0 was used to manage data, transform effect sizes, and calculate overall effect sizes, significance, and effect measure modification by trimester. Available data were divided into three separate data files: Affective Disorders, Personality Disorders, and Psychotic Disorders. Overall effects were analyzed for each psychopathology grouping. Furthermore, specific trimester effects were obtained by classifying “trimester” as an effect measure modifier. Studies without available trimester data were included in the overall effects analyses for each psychopathology grouping, but not in the trimester specific analyses. Analyses were conducted separately for each psychopathology grouping so that trimester effects specific to certain domains of psychopathology could be identified (Figures 2-4). Three psychopathology groupings included the following diagnoses: 1) affective disorders (including unipolar depression, bipolar depression, dysthymia, and affective psychosis), 2) personality disorders (comprised of ASPD and schizoid personality), and 3) psychotic disorders (consisting of schizophrenia, schizoid personality disorder, and affective psychosis). A fixed effect meta-analysis was carried out as we retained the assumption that all included studies estimated the same underlying parameter. All effect sizes in our analyses were expressed as risk ratios (RR). If published effect sizes were not in RR format, they were transformed into RRs using CMA software.

Risk for affective disorders by famine exposure was based upon two previous studies: Brown et al. [15], whose primary outcomes included bipolar disorder and unipolar depression, and Brown and Susser [14], which focused on affective psychosis and neurotic depression. Our assessment of risk for psychotic disorders by famine exposure included four studies, which assessed risk across schizophrenia, schizoid personality disorder, and affective psychosis. Finally, risk for personality disorders by famine exposure included two previous studies, which assessed risk for ASPD and schizoid personality disorder. Timing of exposure was explored as an effective measure modifier across all three domains.

## 7. Results

Overall, we did not suspect selective reporting or publication bias across studies included in each domain. We did not observe any important clinical diversity based on a weighted fail-safe N test.

### 7.1 Affective disorders

To test for heterogeneity among studies of affective disorders, the I^2^ statistic was calculated (I^2^=0, 0, and 15.3 for 1^st^, 2^nd^, and 3^rd^ trimester exposure respectively; see Table 1). The 3^rd^ trimester was the only relevant trimester for affective disorders and had acceptable between-study heterogeneity. No notable influence on risk for affective disorders was observed among offspring of mothers exposed to famine during the 1^st^ trimester (RR=1.08, 95% CI=0.84, 1.35, *p*=0.06). In contrast, a significant increased risk for affective disorders was observed among those who were exposed in both the 2^nd^ trimester (RR=1.45, 95% CI=1.22, 1.72, *p*<0.0001) and 3^rd^ trimester (RR=1.33, 95% CI=1.13, 1.57, *p*=0.0001). However, readers should note that the magnitude of the relative risk for 2^nd^ and 3^rd^ trimester exposure was modest (RR=1.45 and 1.33, respectively).

### 7.2 Psychotic disorders

There was no notable heterogeneity (I^2^ <0.0001) for psychotic disorders for either 2^nd^ or 3^rd^ trimester exposure (See Table 2). Moreover, significant heterogeneity was observed both for 1^st^ trimester exposure and when the timing of exposure was not considered (I^2=^59.66 and 97.99, respectively). As expected, overall heterogeneity was observed in this model (I^2=^91.46).

We observed a significant effect for risk of psychotic disorders across all three trimesters of exposure to famine *in utero*. Specifically, we found an increased risk of psychotic disorders in adulthood among those exposed *in utero* during the 1^st^ trimester (RR=1.46, 95% CI=1.08, 1.97, *p*=0.014), 2^nd^ trimester (RR=1.46, 95% CI=1.13, 1.89, *p*=0.004), and 3^rd^ trimester (RR=1.47, 95% CI=1.10, 1.97, *p*=0.01). The overall risk for psychotic disorders, including studies that did not have trimester data available, was also significant (RR=1.54, 95% CI=1.44, 1.66, *p*<0.001). The relative risks across all three trimesters and overall are modest, ranging from RR=1.46 to RR=1.54.

### 7.3 Personality disorders

No important heterogeneity among studies was noted (I^2^<0.001, <0.001, and 0.07 for 1^st^, 2^nd^, and 3^rd^ trimester exposure, respectively; see Table 3). There was a two-fold increased risk of personality disorders in adulthood among offspring exposed during their 1^st^ trimester (RR=2.31, 95% CI=1.36, 3.92, *p*=0.002). No significant increase in risk for personality disorders among those exposed in either the 2nd or 3^rd^ trimesters was observed.

## 8. Discussion

To our knowledge, this is the first meta-analysis to examine long-term psychopathological effects of nutrient deficiency *in utero* using famine exposure in human populations. We found that those exposed to famine were at significantly increased risk of developing psychological problems in adulthood across all three different domains of psychopathology: affective disorders, psychotic disorders, and personality disorders. The effect of timing of exposure on risk appeared to vary by domain.

In the affective disorders domain, we found a significant increased risk of the disorder following prenatal exposure to famine in the 2^nd^ and 3^rd^ trimesters, but not in the 1^st^ trimester. This finding may be attributed to the maturation/development of the fetal brain systems, especially the central nervous system, during mid-late gestation. The 1^st^ trimester is typically associated with a wide range of health problems in adulthood and viewed as possibly the most vulnerable window of susceptibility in the field. It is important to note that disorders related to neurodevelopment have been identified in offspring exposed in mid-late gestation [23]. It is possible during a period of maturation/development (2^nd^ and 3^rd^ trimester), malnutrition could have an impact over global emotion regulation, which may then lead to an increased propensity for affective disorders. Readers should evaluate our findings with affective disorders with caution since the effect sizes, while significant, were quite modest for 2^nd^ and 3^rd^ trimester exposure.

In the psychotic disorders domain, offspring exposed during each trimester were at a significantly increased risk of developing a psychotic disorder. These findings were not consistent with previous research which showed that psychotic symptoms in adulthood are associated specifically with exposure in early pregnancy. Interestingly, when affective psychosis was removed from the model, a significant effect was only found in the 1^st^ trimester. It is possible that affective psychosis may be more developmentally similar to affective disorders than psychotic disorders. Our inclusion of affective psychosis in this model may explain the discrepancy between our findings and the trimester-specific findings of previous researchers [24, 25].

Consistent with previous research, we found that adults exposed during their 1^st^ trimester were at a significantly increased risk of developing a personality disorder. However, no significant risk was found across ASPD in the overall model, or within those exposures during the 2^nd^ trimester, contrary to previous findings [22]. Over a two-fold increased risk of developing a personality disorder following 1^st^ trimester exposure was observed. Furthermore, the effect size for 1^st^ trimester exposure in this domain was substantially larger than the effect sizes observed in the affective and psychotic domains. This finding indicates that the magnitude of the effect of prenatal famine is seen most dramatically in the personality disorder domain, but further research into the etiology of personality disorders in relation to *in utero* development is needed to provide context for our observation.

Our findings lend further support to the trimester-specific fetal programming theory. We found a unique pattern of relative risks in each domain, suggesting that critical time windows during pregnancy may determine different levels of risk for specific mental health problems in the offspring. Exact mechanisms for this increase in risk cannot be determined in cohort studies, but several potential mechanisms can be explored. It is possible that *in utero* exposure to famine increases risk of psychopathology in adulthood due to a deficiency in specific micronutrients such as iron, protein, and/or folic acid, which are essential for proper neurodevelopment [22]. Increases in RR may also be due to general nutritional deficits from caloric restriction rather than to a deficit in a particular micronutrients. Further research in more controlled studies is necessary to make this distinction.

Despite its powerful indications, trimester-specific relative risk associated with famine exposure should not be interpreted as “causal” at this time. A greater understanding of the mechanisms underlying this association is needed before such an assertion can be made. It is possible that unmeasured confounders contributed to elevated risk for these disorders following famine exposure. For example, there is evidence that tulip bulbs and other toxic food substitutes were consumed during the Dutch Hunger Winter during the height of the famine [26]. Ingesting toxic substances during pregnancy could have similarly negative consequences for fetal brain development. As there were studies utilizing this cohort in all three of the domains, part of the effect under investigation could be explained by toxic food substitutes. In addition, it is likely that the famine itself increased stress responses in pregnant women, leading to elevated cortisol levels, which could also influence the development of the fetus [27]. There was also a rise in infant infections (e.g., tuberculosis, typhoid, dysentery, etc.) after famine [26]. Health complications during infancy can contribute to increased risk for mental disorders later on in life.

Clinically, our findings may be of particular interest to medical professionals and pregnant women, not only in nations where famine is an imminent risk, but worldwide. There are a number of reasons why women may not consume an adequate number of calories during pregnancy, which could mimic the restricted caloric intake observed in famine research. Persistent nausea, dietary restrictions, and financial burdens are common obstacles to sufficient nutrition during pregnancy. Body image disturbances related to pregnancy weight gain may also contribute to dieting behaviors and malnutrition. While famine itself is relatively rare in developed nations, eating disorders have become increasingly common in recent decades and can mimic the starvation associated with famine [28]. Women with a history of disordered eating or body image disturbances may be particularly vulnerable to restricted calorie diets while pregnant, which may in turn have negative consequences for the fetus.

Future research should take advantage of the strengths of animal and human models. Animal models may be more conducive to translational research into the mechanisms that underlie the associations between the distinct timing of exposure and the risk of distinct mental disorders. As previously discussed, ethical constraints do not allow us to conduct controlled experiments with malnutrition in pregnant humans. In animal models, researchers are able to control for confounding factors such as health problems in pregnant animals, ingestion of non-food substitutes, genetic predisposition for certain behaviors, and postnatal environmental factors. Moreover, animal research with rodents would allow us to observe potential effects across multiple generations, as rodent lifespans are much shorter than human lifespans [29]. Identifying any heritable permanent genetic alterations related to prenatal malnutrition would help move the field forward.

There are a number of limitations that should be addressed in our study. First, while multiple studies by different groups of investigators were conducted, they were all based on two cohorts due to the limited number of populations that have been exposed to famine and assessed for psychopathology in adulthood. It is possible that our results would have been different had there been a wider range of populations and backgrounds represented. However, it is notable that the two cohorts included are from vastly different cultural, geographic, and economic backgrounds. An additional limitation is the inclusion of only 6 articles in our meta-analysis, which may restrict the breadth of our interpretations. However, this number of articles was deemed appropriate, due to the statistical power afforded by the rather large sample sizes. Another limitation of this study is that some articles from which data was extracted supplied information about multiple disorders. As these disorders were run separately in the analyses, it is possible that any error attributed to one disorder could also be present in the second disorder. Furthermore, there are a small number of researchers who have published on this topic, some of whom have published dozens of articles that have informed the field. Despite these limitations, our findings contribute to the growing literature in the field of fetal programming. This meta-analysis underscores the need for further research in this specific field, and investment in resources to develop best practices to curtail prenatal undernutrition.

## Figures and Tables

**Table 1:** Risk Ratio (RR) of Affective Disorders by Trimester-specific Famine Exposure.

Timing of Exposure	# of Disorders	RR	95%CI	Z Value	P Value	I^2^
1^st^ Trimester	4	1.06	[0.84-1.35]	0.52	0.61	<.0001
2^nd^ Trimester	4	1.45	[1.22-1.72]	4.16	<.0001	<.0001
3^rd^ Trimester	4	1.33	[1.13-1.57]	3.37	.0001	15.28
Overall	--	1.31	[1.18-1.46]	4.96	<.0001	

RR = Risk Ratio

CI= confidence interval

**Table 2:** Risk Ratio (RR) of Psychotic Disorders by Trimester-specific Famine Exposure.

Timing of Exposure	# of Disorders	RR	95%CI	Z Value	P Value	I^2^
1^st^ Trimester	3	1.46	[1.08 - 1.97]	2.45	0.014	59.66
2^nd^ Trimester	3	1.46	[1.13 - 1.89]	2.89	0.004	<0.0001
3^rd^ Trimester	1	1.47	[1.10 - 1.97]	2.57	0.010	<0.0001
Not specified	3	1.57	[1.44 - 1.70]	10.77	<0.0001	97.99
Overall	--	1.54	[1.44 - 1.66]	11.68	<0.0001	91.55

RR= Risk Ratio

CI= confidence interval

**Table 3:** Risk Ratio (RR) of Personality Disorders by Trimester-specific Famine Exposure.

Timing of Exposure	# of Disorders	RR	95%CI	Z Value	P Value	I^2^
1^st^ Trimester	2	2.31	[1.36-3.92]	3.10	0.002	<.0001
2^nd^ Trimester	2	1.63	[0.96-2.74]	1.82	0.07	0.07
3^rd^ Trimester	1	1.10	[0.42-2.86]	0.20	0.85	<0.0001
Overall	--	1.80	[1.27-2.59]	3.31	0.0001	<0.0001

RR= Risk Ratio

CI= confidence interval
